# Perceptions, Satisfaction, and Barriers to Telemedicine Use: A Community-Based Study From Jeddah, Saudi Arabia

**DOI:** 10.7759/cureus.40738

**Published:** 2023-06-21

**Authors:** Abdullah T Albaghdadi, Manal M Al Daajani

**Affiliations:** 1 Saudi Board of Preventive Medicine Program, Public Health Administration, Ministry of Health, Jeddah, SAU; 2 Public Health Department, Jeddah Health Affairs, Jeddah, SAU

**Keywords:** saudi arabia, barriers, satisfaction, perceptions, telehealth, telemedicine

## Abstract

Background

Telemedicine has expanded significantly, driven by technology and the necessity for accessible healthcare. However, users’ knowledge, attitudes, and perceived barriers determine its application. This study aimed to assess these factors among patients in Jeddah, Saudi Arabia.

Methodology

We conducted a cross-sectional study on 403 participants from Ministry of Health centers in Jeddah from February to May 2023. A structured questionnaire was used for data collection, and subsequent analysis was performed using SPSS version 28.0 (IBM Corp., Armonk, NY, USA).

Results

Most participants (93.1%) agreed that telehealth services have improved healthcare accessibility and expressed willingness to participate in future telemedicine consultations. However, 73.7% felt potential embarrassment or discomfort due to camera and equipment presence. Remarkably, 76.2% of participants believed telemedicine suits all medical conditions, and 95% recommended its use. Barriers to telemedicine use were identified, including the need to travel to access healthcare services in the absence of telemedicine and the associated inconvenience and cost. The overall satisfaction score was 4.56 ± 0.78, with the highest satisfaction reported for the ability to talk freely over telemedicine (4.64 ± 0.76) and the ease of registration/scheduling (4.63 ± 0.82). Significant differences (p < 0.001) in satisfaction scores were found across various age groups, gender, nationality, employment status, and education level. Patients aged >55 years and those who used telemedicine services for the first time were associated with a significantly increased risk of poor satisfaction (odds ratio (OR) = 8.068, p = 0.011 and OR = 8.919, p = 0.005, respectively).

Conclusions

The findings suggest high satisfaction and positive attitudes toward telemedicine services in Jeddah, Saudi Arabia, despite identifiable barriers such as potential discomfort from camera presence. Patient age and familiarity with telemedicine services significantly influenced satisfaction levels, indicating areas that require attention for the successful implementation and expansion of telemedicine in Saudi Arabia.

## Introduction

The rapid technological advancements of the twenty-first century have dramatically impacted various sectors, including healthcare. Telemedicine, defined as the delivery of healthcare services using telecommunication technologies, is one of the remarkable developments in this era [1]. These services encompass a range of applications, such as remote patient monitoring, virtual consultations, and mobile health apps, all aiming to augment the accessibility and quality of healthcare [2-4].

Telemedicine has surged to the forefront of healthcare delivery as we continue to navigate the challenges posed by the coronavirus disease 2019 (COVID-19) pandemic [5,6]. The need for social distancing measures and the aim of protecting vulnerable patients necessitated a rapid shift toward digital health services, making telemedicine an invaluable tool in the fight against the virus [7-9].

Moreover, telemedicine holds the promise of tackling some of the longstanding challenges in healthcare. It potentially offers solutions to geographical barriers, inequitable access to specialized services, and escalating healthcare costs [1,10]. The benefits of telemedicine are particularly salient in countries with vast rural areas or limited health infrastructure [11], such as Saudi Arabia, where ensuring comprehensive healthcare coverage can be logistically challenging [12].

Despite these potential benefits, the implementation of telemedicine faces numerous barriers. Concerns regarding privacy and data security, a lack of patient and provider familiarity with the technology, and infrastructural constraints can impede the adoption of telemedicine services [13-15]. Understanding these barriers is crucial to devise effective strategies to increase the acceptance and utilization of telemedicine.

However, while there is a growing body of international literature on telemedicine, there is a relative scarcity of research focusing on the Middle Eastern context, particularly Saudi Arabia. This study aimed to fill this gap by assessing the satisfaction, knowledge, and attitudes toward telemedicine services among the Saudi population in Jeddah, who visited the primary healthcare centers (PHCs) of the Ministry of Health (MOH).

## Materials and methods

Study design and setting

This analytical, community-based, cross-sectional study was conducted from February to May 2023. The study took place in PHC telemedicine services provided by the MOH in Jeddah, Saudi Arabia. This study received approval from the Local Committee for Research Ethics in Jeddah (IRB: A01543) and was conducted in concordance with the Declaration of Helsinki, the Good Clinical Practice (GCP) code, and local regulations. The study did not involve any medicinal products or devices; hence, there was no anticipated risk of unexpected adverse events. The confidentiality of participants was maintained by collecting data anonymously. Consent was obtained implicitly from participants who agreed to complete the questionnaire, including a statement about the study’s objectives and methodologies.

Study participants

Patients aged ≥18 years, both male and female, who participated in the telemedicine services of MOH before data collection and agreed to participate in the study were included. Participants from private clinics and hospitals were excluded from the study.

Variables

The primary variables of the study were participants’ satisfaction, knowledge, and attitude toward telemedicine services, which were evaluated using a structured questionnaire. The secondary variables included demographic characteristics such as age, sex, nationality, experience with telemedicine networks, education level, occupation, and reason for using telemedicine.

Data sources and measurement

Data were collected in the PHC during clinical visits by volunteers trained for this purpose. Data were collected through a structured questionnaire that has been previously validated in Arabic and English [16,17]. Volunteer data collectors were trained and assigned to gather data from the participants. The questionnaire was divided into four parts, namely, demographic data, knowledge and attitude assessment, satisfaction rating, and assessment of obstacles to using telemedicine.

Sampling method and sample size

Multicenter cluster sampling was employed. The study area was divided into five hospitals governing 37 PHC telemedicine providers in Jeddah, Saudi Arabia. From each hospital, PHCs were selected using a simple random sampling technique. From each PHC, participants were selected using a convenience sampling technique. The sample size was determined using G*Power software version 3.1.9.7 for Windows. Based on an overall satisfaction rate with telemedicine of 74.3 ± 7.78 from a previous study [16], with a precision of 80%, a margin of error of 5%, and a 95% confidence interval (CI), a total of 364 participants were required. To account for an expected rate of incomplete response of 10%, we included 403 participants.

Statistical analysis

Data from the study were entered into an Excel sheet for data cleaning and coding. Statistical analysis was performed using SPSS version 28.0 for Windows (IBM Corp., Armonk, NY, USA). Continuous data were presented as mean (standard deviation). Categorical data were summarized using numbers and percentages. The chi-square test was used to assess the association between satisfaction and categorical variables, while the Student’s t-test and analysis of variance test, with post hoc analysis using the Tukey test, were used to evaluate this association with continuous variables. A multivariable logistic model was constructed to identify independent predictors of poor satisfaction. Odds ratio (OR) with a 95% CI was used to represent the degree of association between dependent and independent variables. A p-value of less than 0.05 was considered statistically significant.

## Results

Demographic characteristics

This study involved 409 participants, of whom 403 (98.5%) completed the questionnaire. Age distribution was as follows: 46.4% (36-45 years), 30% (26-35 years), 9.7% (18-25 years), 7.4% (46-55 years), and 6.5% (>55 years). The sample comprised 60.8% males (n = 245) and 39.2% females, with 86.1% being Saudi nationals. Regarding employment, 47.9% were in the governmental sector, 20.8% were unemployed, and 17.9% were in the private sector, with 7.2% students and 6.2% retirees. Education levels were as follows: college/university (33.50%), high school (29.28%), postgraduate (26.05%), intermediate (3.72%), primary (5.46%), and illiterate (1.99%). Chronic disease prevalence was 40.9%, while 57.1% reported no such conditions. Table 1 summarizes the demographic characteristics of included participants.

**Table 1:** Demographic characteristics of included participants.

Parameters		N (%)
Age (years)	26–35	121 (30.02%)
	36–45	187 (46.40%)
	46–55	30 (7.44%)
	18–25	39 (9.68%)
	>55	26 (6.45%)
Gender	Male	245 (60.79%)
	Female	158 (39.21%)
Nationality	Saudi	347 (86.10%)
	Non-Saudi	56 (13.90%)
Employment status	Unemployed	84 (20.84%)
	Private sector	72 (17.87%)
	Governmental sector	193 (47.89%)
	Student	29 (7.20%)
	Retired	25 (6.20%)
Education level	College/University	135 (33.50%)
	High school	118 (29.28%)
	Intermediate	15 (3.72%)
	Primary	22 (5.46%)
	Postgraduate	105 (26.05%)
	Illiterate	8 (1.99%)
Do you have any chronic diseases? e.g., diabetic/hypertension	No	230 (57.07%)
	Yes	165 (40.94%)

Previous experience with telemedicine and its technologies

The study further explored participants’ experiences with telemedicine technologies. Of all respondents, 16.6% had never used telemedicine, while 52.6% had started using it before the COVID-19 pandemic and 30.9% after the emergence of the pandemic. The reasons for using telemedicine varied, with 48.21% of the participants citing emergencies, 31.25% for lockdown, 16.96% for adverse drug events, 9.23% for exacerbated chronic conditions, 27.08% for medical consultations, 14.88% for follow-ups, and 2.68% for other reasons. Regarding devices used, 39.95% regularly used phone or video calls, 20.60% used live video conferences, 15.38% used laptops, 7.94% used personal computers, and 47.39% used some of these devices. A negligible proportion (1.49%) reported not using any of these devices. When asked about their knowledge of types of telemedicine, 53.35% knew about live video conferencing, 41.19% about store-and-forward asynchronous video, 35.73% about remote patient monitoring (RPM), 40.69% about mobile health or mHealth, and 3.97% did not know about any of them. Regarding preferred telemedicine services, 33.25% preferred live video conferencing, 42.93% preferred store-and-forward asynchronous video, 33.75% preferred RPM, 42.43% preferred mHealth, while 7.94% did not have a preference, as shown in Table 2.

**Table 2:** Previous experience with telemedicine and its technologies.

Questions		N (%)
When was the first time you used telemedicine?	I haven’t used it at all	67 (16.63%)
	Before the emergence of COVID-19	212 (52.61%)
	After the emergence of COVID-19	124 (30.77%)
If yes, please choose the reasons for using telemedicine	Emergency	162 (40.20%)
	Lockdown	105 (26.05%)
	Drug adverse events	57 (14.14%)
	Exacerbated chronic condition	31 (7.69%)
	Medical consultation	91 (22.58%)
	Follow-up	50 (12.41%)
	Others	9 (2.23%)
I use these devices regularly	Phone or video calls	161 (39.95%)
	Live video conferences	83 (20.60%)
	Laptops	62 (15.38%)
	Personal computers	32 (7.94%)
	Some of these devices	191 (47.39%)
	None of these devices	6 (1.49%)
Which type of telemedicine do you know?	Live video conferencing	215 (53.35%)
	Store-and-forward synchronous video	166 (41.19%)
	Remote patient monitoring	144 (35.73%)
	Mobile health or mHealth	164 (40.69%)
	I do not know any of them	16 (3.97%)
Which type of telemedicine service do you prefer?	Live video conferencing	134 (33.25%)
	Store-and-forward synchronous video	173 (42.93%)
	Remote patient monitoring	136 (33.75%)
	Mobile health or mHealth	171 (42.43%)
	I do not know any of them	32 (7.94%)

Perspectives toward telemedicine

In response to questions about perceptions of telemedicine, most participants held favorable views. A substantial 64.5% strongly agreed that telemedicine saves time, and 64.3% (n = 259) strongly agreed it is necessary for patient care. The importance of telemedicine in rural and underserved areas was strongly supported by 67.7% of respondents. The belief that telemedicine can save effort and money was strongly endorsed by 68.5% and 65.5% of participants, respectively. Similarly, 67.2% strongly agreed that it could reduce transportation costs. However, regarding its potential to decrease medical mistakes, a majority (56.1%) strongly disagreed. The majority (67.5%) strongly agreed that telemedicine could reduce waiting lists at medical centers, and 64.8% strongly agreed that it could enhance doctor-patient relationships. In the case of providing appropriate emergency instructions, 64.3% strongly agreed. Regarding privacy concerns, 58.6% strongly disagreed that telemedicine could jeopardize patient privacy, and 60.8% strongly disagreed that it could lead to unauthorized disclosure of medical information. On the other hand, a significant proportion of participants (73.7%) agreed that the presence of a camera and other equipment could make them feel uncomfortable or embarrassed. An overwhelming 93.1% agreed that telehealth services made receiving healthcare easier, and 76.2% believed telemedicine was suitable for all medical conditions. as shown in Table 3.

**Table 3:** Perspectives toward telemedicine.

Question	Answer	Total
Do you think that telemedicine will save time?	Strongly disagree	10 (2.48%)
	Disagree	14 (3.47%)
	Neither agree nor disagree	36 (8.93%)
	Agree	83 (20.60%)
	Strongly agree	260 (64.52%)
Do you think that telemedicine is necessary for patient care?	Strongly disagree	5 (1.24%)
	Disagree	16 (3.97%)
	Neither agree nor disagree	44 (10.92%)
	Agree	79 (19.60%)
	Strongly agree	259 (64.27%)
Do you think that telemedicine is necessary for rural areas and areas that suffer from a shortage of healthcare services?	Strongly disagree	9 (2.23%)
	Disagree	18 (4.47%)
	Neither agree nor disagree	25 (6.20%)
	Agree	78 (19.35%)
	Strongly agree	273 (67.74%)
Do you think that telemedicine will save effort?	Strongly disagree	5 (1.24%)
	Disagree	10 (2.48%)
	Neither agree nor disagree	32 (7.94%)
	Agree	80 (19.85%)
	Strongly agree	276 (68.49%)
Do you think that telemedicine will save money?	Strongly disagree	5 (1.24%)
	Disagree	10 (2.48%)
	Neither agree nor disagree	38 (9.43%)
	Agree	86 (21.34%)
	Strongly agree	264 (65.51%)
Do you think that telemedicine will decrease transportation costs?	Strongly disagree	7 (1.74%)
	Disagree	9 (2.23%)
	Neither agree nor disagreed	29 (7.20%)
	Agree	87 (21.59%)
	Strongly agree	271 (67.25%)
Do you think that telemedicine will decrease medical mistakes?	Strongly disagree	226 (56.08%)
	Disagree	57 (14.14%)
	Neither agree nor disagree	30 (7.44%)
	Agree	61 (15.14%)
	Strongly agree	29 (7.20%)
Do you think that telemedicine will decrease waiting lists at medical centers?	Strongly disagree	8 (1.99%)
	Disagree	19 (4.71%)
	Neither agree nor disagree	27 (6.70%)
	Agree	77 (19.11%)
	Strongly agree	272 (67.49%)
Do you think that telemedicine will improve the doctor-patient relationship?	Strongly disagree	10 (2.48%)
	Disagree	18 (4.47%)
	Neither agree nor disagree	30 (7.44%)
	Agree	84 (20.84%)
	Strongly agree	261 (64.76%)
Do you think that telemedicine can help in giving the patient appropriate instructions in emergency situations?	Strongly disagree	6 (1.49%)
	Disagree	15 (3.72%)
	Neither agree nor disagree	33 (8.19%)
	Agree	90 (22.33%)
	Strongly agree	259 (64.27%)
Do you think that telemedicine can put the patient’s privacy at risk?	Strongly disagree	236 (58.56%)
	Disagree	57 (14.14%)
	Neither agree nor disagree	51 (12.66%)
	Agree	22 (5.46%)
	Strongly agree	37 (9.18%)
Do you think that telemedicine may lead to the disclosure of patients’ medical information to unauthorized persons?	Strongly disagree	245 (60.79%)
	Disagree	43 (10.67%)
	Neither agree nor disagree	60 (14.89%)
	Agree	25 (6.20%)
	Strongly agree	30 (7.44%)
Do you think that telemedicine is suitable for all medical conditions?	Yes	307 (76.18%)
	No	96 (23.82%)
Do you think telehealth services have made receiving healthcare easier today?	Agree	375 (93.05%)
	Disagree	28 (6.95%)
Do you think the presence of the camera and other equipment can embarrass you or make you feel uncomfortable?	Agree	297 (73.70%)
	Disagree	106 (26.30%)

**Figure 1:**
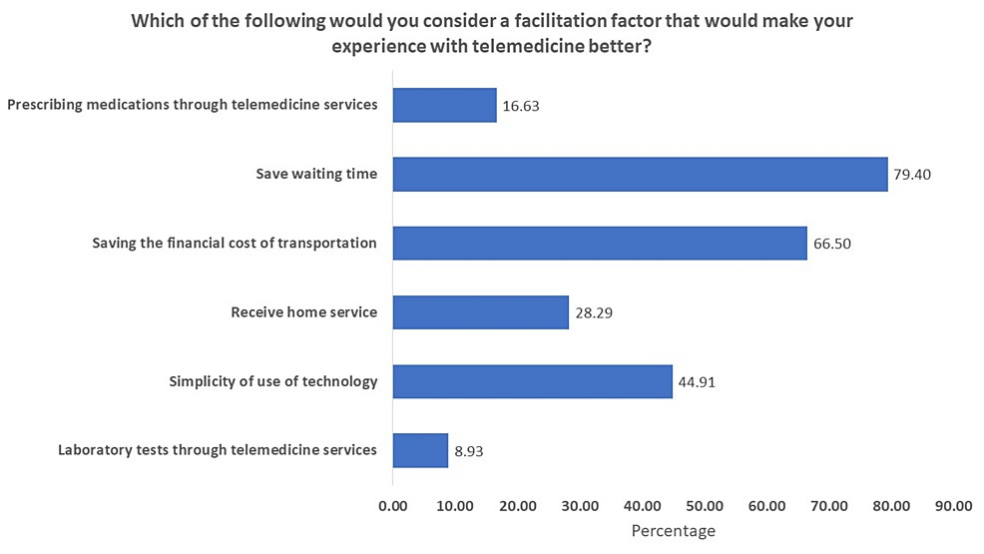
Factors that may enhance the experience of telemedicine.

When they were asked about the factors that may facilitate their experience with telemedicine and make it better, the majority (79.40%) chose “saving waiting time,” followed by “saving the financial costs of transportation” (66.50%), “simplicity use of technology” (44.91%), and “receiving home service” (28.29%), as shown in Figure 1.

Attitudes toward telemedicine

The participants’ attitudes toward telemedicine were generally positive. Additionally, 86.4% indicated they might have to miss work or other obligations to see a therapist if telehealth services were not available. In the absence of telemedicine, most participants would have needed to travel between 15 and 30 minutes (44.9%) or 30 and 60 minutes (31.8%) to receive care. Some consequences of this included losing time from work (24.07%) or incurring other expenses (7.44%). If telemedicine had not been an option, the majority would have either driven to see a specialist face-to-face (37.72%) or contacted their local clinic (31.51%). A vast majority (93.1%) of the participants expressed willingness to participate in another telemedicine consultation. Lastly, a significant 95% of the participants would recommend telemedicine, as shown in Table 4.

**Table 4:** Attitudes toward telemedicine.

Question	Answers	N (%)
In case you need healthcare, do you think you might have to miss work/get things done to see a therapist if telehealth services are not available?	Agree	348 (86.35%)
	Disagree	55 (13.65%)
If telemedicine had not been available for your consult today, how far would you have had to travel to receive care?	Less than 15 minutes	70 (17.37%)
	15–30 minutes	181 (44.91%)
	30–60 minutes	128 (31.76%)
	2 hours	13 (3.23%)
	More than 2 hours	11 (2.73%)
If telemedicine had not been available and you had to travel to meet face-to-face with the provider to receive care, which of the following would apply? (please check all that apply)	I would have lost time at work	97 (24.07%)
	My companions would have lost time at work	61 (15.14%)
	I would have paid for meals while I was away from home	1 (0.25%)
	I would have paid for a hotel to spend the night	5 (1.24%)
	Other expenses (please specify)	30 (7.44%)
If telemedicine had not been available for your consult today, which of the following would have been your alternative plan of action?	I would have driven to see the specialist face-to-face	152 (37.72%)
	I would have contacted my local clinic to see if they could assist	127 (31.51%)
	I wouldn’t go see any doctor	19 (4.71%)
	The use of alternative medicine (honey, nigella, Indian installment, etc.)	9 (2.23%)
Would you be willing to participate in another telemedicine consultation?	Yes	375 (93.05%)
	No	18 (4.47%)
	Not sure	10 (2.48%)
Do you recommend telemedicine?	Yes	383 (95.04%)
	No	20 (4.96%)

**Figure 2:**
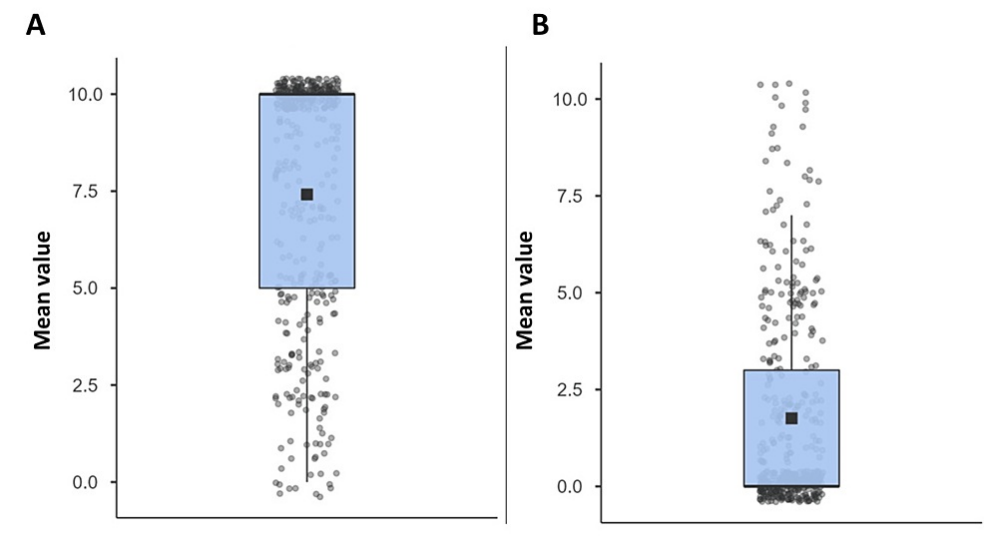
The level of preference and difficulty of telemedicine. (A) Participants’ preference for using telemedicine over the traditional way (mean score out of 10). (B) Difficulty in using telemedicine (mean score out of 10).

When participants were asked to evaluate their preference for using telemedicine instead of the traditional way, the mean score was 7.41 ± 3.23 out of 10. Regarding the difficulty of using telemedicine, they rated it as 1.76 ± 2.58 out of 10, as shown in Figure 2.

Satisfaction

The overall satisfaction score was 4.56 ± 0.78, indicating a high level of satisfaction among the study participants. The aspect with the highest satisfaction score was the ability to talk freely over telemedicine (4.64 ± 0.76), closely followed by ease of registration/scheduling (4.63 ± 0.82) and understanding of the recommendations or diagnosis made (4.63 ± 0.76), as shown in Table 5.

**Table 5:** Satisfaction domains across the included populations.

Satisfaction		Very Satisfied	Satisfied	Neutral	Dissatisfied	Very dissatisfied	Overall satisfaction score
How would you rate your telemedicine consultation on these factors?	Ease of registration/scheduling	315 (78.16%)	47 (11.66%)	24 (5.96%)	11 (2.73%)	5 (1.24%)	4.63 ± 0.82
	Quality of the visual image	296 (73.45%)	51 (12.66%)	33 (8.19%)	18 (4.47%)	3 (0.74%)	4.54 ± 0.88
	Quality of the audio sound	307 (76.18%)	43 (10.67%)	31 (7.69%)	17 (4.22%)	3 (0.74%)	4.58 ± 0.87
	Ability to talk freely over telemedicine	307 (76.18%)	46 (11.41%)	32 (7.94%)	8 (1.99%)	2 (0.50%)	4.64 ± 0.76
	Ability to understand the recommendations or diagnosis made	303 (75.19%)	57 (14.14%)	31 (7.69%)	6 (1.49%)	3 (0.74%)	4.63 ± 0.76
	The comfort of the telemedicine suite (the location where I received my care)	300 (74.44%)	53 (13.15%)	35 (8.68%)	9 (2.23%)	3 (0.74%)	4.59 ± 0.80
	The overall quality of care provided	294 (72.95%)	49 (12.16%)	41 (10.17%)	13 (3.23%)	2 (0.5%)	4.55 ± 0.84
	Overall telemedicine consult experience	299 (74.19%)	42 (10.42%)	43 (10.67%)	16 (3.97%)	3 (0.74%)	4.53 ± 0.89
Overall satisfaction score		4.56 ± 0.78					

Regarding demographics, significant differences (p < 0.001) in satisfaction scores were found across various age groups, gender, nationality, employment status, education level, and the timing of the first telemedicine use. Participants aged 36-45 years reported the highest satisfaction score (4.86 ± 0.44), whereas those older than 55 years reported the lowest (3.63 ± 1.20). Males exhibited higher satisfaction (4.73 ± 0.68) than females (4.29 ± 0.85). Saudis had higher satisfaction scores (4.62 ± 0.75) compared to non-Saudis (4.18 ± 0.89). Participants employed in the governmental sector expressed the highest satisfaction (4.83 ± 0.50), whereas unemployed individuals reported the lowest (3.99 ± 0.94). Postgraduates had the highest satisfaction (4.87 ± 0.42), whereas those with primary education had the lowest (4.05 ± 1.21). Participants who used telemedicine after the emergence of COVID-19 exhibited the highest satisfaction (4.88 ± 0.40), whereas those who had never used it reported the lowest satisfaction (3.93 ± 1.08), as shown in Table 6.

**Table 6:** Associations between demographics and satisfaction. The different letters (a, b, c, d, and e) assigned to each cell within the same question represent homogeneous subsets of data, as determined by post hoc Tukey’s test following the analysis of variance test. Cells within the same question that carry the same letter do not significantly differ (p > 0.05). Conversely, cells carrying different letters are statistically distinct (p < 0.05). Each unique letter represents a subset of data that is significantly different from the others.

Parameters		Mean ± SD	P-value
Age (years)	26–35	4.48 ± 0.80^a^	<0.001
	36–45	4.86 ± 0.44^b^	
	46–55	4.06 ± 0.96^c,d^	
	18–25	4.35 ± 0.74^a,c^	
	>55	3.63 ± 1.20^d^	
Gender	Male	4.73 ± 0.68	<0.001
	Female	4.29 ± 0.85	
Nationality	Saudi	4.62 ± 0.75	<0.001
	Non-Saudi	4.18 ± 0.89	
Employment status	Unemployed	3.99 ± 0.94^a^	<0.001
	Private sector	4.58 ± 0.77^b,e^	
	Governmental sector	4.83 ± 0.50^c,b^	
	Student	4.67 ± 0.57^b,d,e^	
	Retired	4.17 ± 1.11^a,e^	
Education level	College/University	4.57 ± 0.77^a^	<0.001
	High school	4.37 ± 0.86^a^	
	Intermediate	4.60 ± 0.64^a,b,c^	
	Primary	4.05 ± 1.21^b^	
	Postgraduate	4.87 ± 0.42^c^	
	Illiterate	4.34 ± 0.71^a,c^	
Do you have any chronic diseases? e.g., diabetic/hypertension	No	4.51 ± 0.77	0.078
	Yes	4.65 ± 0.76	
When was the first time you used telemedicine?	I haven’t used it at all	3.93 ± 1.08^a^	<0.001
	Before the emergence of COVID-19	4.34 ± 0.80^b^	
	After the emergence of COVID-19	4.88 ± 0.40^c^	

Predictors of poor satisfaction

Univariate analysis revealed that age, gender, employment status, education level, and the timing of first telemedicine use significantly influenced satisfaction levels. Individuals aged between 46 and 55 years and those older than 55 years were most likely to express dissatisfaction (OR = 3.53, 95% CI = 1.12 to 11.11; p = 0.031 and OR = 7.47, 95% CI = 2.54 to 22.03; p < 0.001, respectively). Additionally, females were more likely to report poor satisfaction compared to males (OR = 2.91, 95% CI = 1.34 to 6.29; p = 0.007). Interestingly, individuals employed in the private and governmental sectors were less likely to report poor satisfaction (OR = 0.34, 95% CI = 0.12 to 0.99; p = 0.049 and OR = 0.12, 95% CI = 0.04 to 0.35; p < 0.001, respectively). Participants who had not used telemedicine before had significantly higher odds of poor satisfaction (OR = 20.09, 95% CI = 5.60 to 72.00; p < 0.001). In the multivariable logistic model, age and the timing of first telemedicine use remained significant predictors. Participants aged 46-55 years and those over 55 years of age were significantly more likely to report poor satisfaction (OR = 7.262, 95% CI = 1.567 to 33.656; p = 0.011 and OR = 8.068, 95% CI = 1.610 to 40.430; p = 0.011, respectively). Individuals who had not used telemedicine before were associated with a significantly higher risk of being dissatisfied compared to those who used it before the COVID-19 pandemic (OR = 8.919, 95% CI = 1.961 to 40.565; p = 0.005), as shown in Table 7.

**Table 7:** Predictors of poor satisfaction.

Predictors		Univariable		Multivariable	
		OR (95% CI)	P-value	OR (95% CI)	P-value
Age (years)	26–35	Reference			
	36–45	0.47 (0.16 to 1.39)	0.170	1.772 (0.479 to 6.559)	0.392
	46–55	3.53 (1.12 to 11.11)	0.031	7.262 (1.567 to 33.656)	0.011
	18–25	0.37 (0.05 to 3.07)	0.358	0.302 (0.030 to 3.000)	0.307
	>55	7.47 (2.54 to 22.03)	<0.001	8.068 (1.610 to 40.430)	0.011
Gender	Male	Reference			
	Female	2.91 (1.34 to 6.29)	0.007	2.053 (0.727 to 5.798)	0.174
Nationality	Saudi	Reference			
	Non-Saudi	2.01 (0.82 to 4.94)	0.127	1.016 (0.318 to 3.250)	0.979
Employment status	Unemployed	Reference			
	Private sector	0.34 (0.12 to 0.99)	0.049	0.862 (0.237 to 3.130)	0.821
	Governmental sector	0.12 (0.04 to 0.35)	<0.001	0.708 (0.173 to 2.895)	0.631
	Student	0.164 (0.021 to 1.30)	0.087	1.212 (0.104 to 14.190)	0.878
	Retired	0.88 (0.26 to 2.93)	0.830	0.439 (0.080 to 2.400)	0.342
Education level	College/University	Reference			
	High school	1.73 (0.71 to 4.21)	0.225	1.132 (0.382 to 3.350)	0.823
	Intermediate	1.00 (0.12 to 8.49)	1.00	1.333 (0.121 to 14.651)	0.814
	Primary	5.25 (1.65 to 16.69)	0.005	3.561 (0.747 to 16.980)	0.111
	Postgraduate	0.13 (0.02 to 1.08)	0.059	0.200 (0.020 to 1.963)	0.167
Do you have any chronic diseases? e.g., diabetic/hypertension	No	Reference			
	Yes	0.98 (0.46 to 2.12)	0.96	0.557 (0.177 to 1.746)	0.315
When was the first time you used telemedicine?	I haven’t used it at all	20.09 (5.60 to 72.00)	<0.001	8.919 (1.961 to 40.565)	0.005
	Before the emergence of COVID-19	Reference			
	After the emergence of COVID-19	7.46 (2.06 to 27.00)	0.002	4.010 (0.965 to 16.663)	0.056

## Discussion

This cross-sectional study aimed to gauge the Saudi population’s attitudes, perspectives, and satisfaction toward telemedicine. Our findings suggest an overwhelmingly positive perception toward telemedicine, demonstrating a strong inclination to embrace this novel method of healthcare delivery. The demographic distribution within the study indicates a dominance of younger age groups (26-45 years), which likely explains the high percentage of participants who have experience with telemedicine (83.4%). This finding aligns with several international studies [10,18], showing a higher propensity among younger populations to use digital health tools. This demographic bias may also explain the reported high satisfaction ratings, as younger cohorts tend to have higher digital literacy.

Interestingly, the majority of participants started using telemedicine before the COVID-19 pandemic, contradicting the global trend where the pandemic has been a catalyst for telemedicine adoption [19]. This suggests that the Saudi healthcare system had already made significant strides in implementing telehealth services pre-pandemic, laying a firm foundation that allowed for easier adaptation during the health crisis [18]. A study by AlDossary et al. highlighted the proactive initiatives by Saudi Arabia’s MOH in encouraging digital health adoption even before the COVID-19 pandemic. This included implementing various telehealth services, which were already well-received by the public, ensuring a firm groundwork that facilitated adaptation during the subsequent health crisis [20]. Previous experiences with pandemics such as Middle East respiratory syndrome may have also encouraged a more proactive stance toward digital health [21].

Concerning the types of telemedicine used, our findings align with global trends. Mobile health (mHealth) and asynchronous telemedicine methods were popular among participants, consistent with global studies emphasizing their convenience and flexibility [22]. This aligns with an investigation by Kao et al., which identified these modalities as the fastest-growing segments in the telehealth industry worldwide, attributed mainly to their convenience, flexibility, and compatibility with a wide range of medical conditions [23]. Our results further corroborate the trend seen in research by Lee et al., which highlighted the surge of mHealth adoption due to its accessibility and the ubiquity of mobile devices, marking a significant shift in healthcare delivery [24]. However, many of our participants were experiencing telemedicine for the first time, consistent with the findings of Singh et al., who also found a gap in public awareness of various telemedicine modalities [25], highlighting the need for increased awareness and education efforts.

The perspectives toward telemedicine were predominantly positive in our cohort. Many respondents agreed on the potential of telemedicine to save time, effort, and money and reduce waiting lists at medical centers. This reflects the global sentiment that telemedicine is a promising solution to healthcare accessibility and efficiency problems [15]. Privacy concerns were also well-addressed in our study, as the majority disagreed that telemedicine could lead to privacy breaches, which contrasts with many international studies suggesting patient apprehension about privacy [26]. This difference could be attributed to cultural factors.

Our findings show high satisfaction with telemedicine services, aligning with local and global trends [5,16,27]. The overall satisfaction score and high scores for specific aspects such as free communication and ease of use indicate successful implementation and promise for further expansion. However, the multivariable logistic model identified older age and no prior use of telemedicine as predictors of poor satisfaction. This highlights areas where targeted interventions could improve overall satisfaction. A US-based study also identified similar factors as barriers to telemedicine satisfaction [28]. Similarly, Abdel Nasser et al. showed that older people in Saudi Arabia preferred face-to-face consultation over telemedicine to express all their concerns verbally and nonverbally [17].

Telemedicine has several advantages and disadvantages. On the positive side, telemedicine has the potential to revolutionize patient care by facilitating access, especially for those in remote or underserved areas, and reducing healthcare costs [1]. The time efficiency of telemedicine, bypassing the need for travel and in-person wait times, is another significant advantage [29]. Conversely, telemedicine poses several challenges. The digital divide, particularly among older adults and rural or low-income populations, may exacerbate health disparities if not carefully addressed [30]. The need for high-speed internet and a certain level of digital literacy also pose barriers to telemedicine access [9].

Current challenges in implementing telemedicine require urgent attention. The stability and security of video call systems are paramount in ensuring patient privacy and fostering trust in telemedicine [31]. Equally important is the mitigation of technological issues that can disrupt the smooth delivery of care. In this context, the integration of high-quality audio and video capabilities, and the assurance of secure data transmission, are crucial for telemedicine’s growth [32].

Emerging technologies, such as satellite systems, can complement telemedicine to create a comprehensive healthcare ecosystem. For example, outpatient appointment systems can streamline patient scheduling, reducing wait times and enhancing healthcare efficiency [33]. Satellite technologies can also support telemedicine in remote areas where internet access is unreliable, expanding the reach of virtual care.

Limitations

While our findings contribute valuable insights to the existing body of knowledge, they should be interpreted in light of several limitations. First, our study’s cross-sectional design precludes the establishment of causality. Additionally, there may be selection bias, as participants with access to and familiarity with online tools were more likely to participate in the study. Moreover, the younger age and higher education level of our participants limit the generalizability of the findings to the broader population. Future studies should aim for more representative samples and explore longitudinal designs.

Recommendations

Our study’s findings call for several recommendations. Despite high satisfaction and positive attitudes toward telemedicine, there is room for improvement. Focused efforts are required to enhance the telemedicine experience for older users and those new to the service. This could be achieved through user-friendly interfaces, personalized training, and dedicated support. Additionally, concerted awareness campaigns are required to familiarize the public with different types of telemedicine services, maximizing their potential benefits. Lastly, while the majority did not express privacy concerns, it remains vital for healthcare providers to maintain robust security measures, reassuring the public of their commitment to patient confidentiality.

## Conclusions

Our study reveals a promising landscape for telemedicine in Saudi Arabia. The positive attitudes, perceptions, and high levels of satisfaction suggest that telemedicine could significantly transform healthcare delivery in the country; however, these findings should be interpreted with caution in view of our study’s limitations. While challenges exist, particularly in engaging older populations and first-time users, targeted strategies can address these issues. The future of telemedicine in Saudi Arabia looks promising, and its continued integration into healthcare systems can potentially drive efficiencies and improve patient outcomes.
